# Metabolism-Mediated Regulation of Brain–Heart Interactions

**DOI:** 10.3390/ijms27093712

**Published:** 2026-04-22

**Authors:** Zemin Liu, Ruiyun Peng, Li Zhao

**Affiliations:** Beijing Institute of Radiation Medicine, Beijing 100850, China

**Keywords:** heart, brain, metabolic, metabolic regulation

## Abstract

Cardiovascular and cerebrovascular diseases are serious threats to human health and impose a significant burden on individuals and society. As the two critical and complex organs with the highest metabolic demands, the brain and the heart form an interactive relationship through metabolic networks. As a core prerequisite for maintaining the normal physiological functions of the body, metabolic homeostasis is also a crucial foundation for ensuring the brain–heart synergy. When the human metabolism is in a stable state, the energy supply and material exchange of the brain and the heart can accurately match demand, the neural signal transmission is smooth, and the myocardial contraction is strong and regular—thus ensuring the coordinated and unified functions of these two vital organs. However, once metabolic homeostasis is disrupted, problems such as energy metabolism disorders will arise, which will then become a core inducing mechanism for cardiovascular and cerebrovascular comorbidities. This article presents a review of the research progress on the potential mechanisms of brain-heart interactions based on metabolic regulation from three aspects: neurometabolic, endocrino-metabolic and immune–metabolic regulation, the impact of cardiac function on brain metabolism, and the bidirectional regulation of brain-heart metabolism.

## 1. Introduction

Cardiovascular and cerebrovascular diseases are major global health threats. In China, cardiovascular and cerebrovascular diseases cause 3.5 million deaths each year, among which myocardial infarction (MI) and cerebral infarction account for more than 80% of all deaths. As two of the most important organs of the human body, the metabolism of the brain and heart is closely related to their functions.

In the 17th century, the connection between the brain and the heart was noted in cases of sudden death due to acute psychological stress [1]. Physicians believe that the influence of the sympathetic nerve, especially the activity of intracardiac ganglia, is the primary cause of heartbeat [2]. In the 1800s, Claude Bernard proposed the concept of the “brain-heart axis”, and studies on brain-heart interactions began to emerge. Subsequent studies have revealed the functional regulation of brain-heart interactions and their potential mechanisms. Studies have shown that various types of stroke can cause cardiac dysfunction by activating the hypothalamic-pituitary-adrenal (HPA) axis and inducing a systemic inflammatory response [3,4]. On the other hand, cardiac injuries can also affect cerebral blood flow and neuron survival. For example, after asphyxial cardiac arrest, cerebral blood flow decreases, and neuronal cell death increases [5]. Furthermore, the stimulation of specific brain regions could trigger sympathetic/parasympathetic nerve responses, thereby affecting cardiac activity [6]. In conclusion, the interactions between the brain and heart play critical roles in maintaining the balance and stability of the body.

With the progress in experimental and medical technologies, scientists have revealed that cardiac functions are regulated by specific brain regions. Stroke can activate inflammatory responses in relevant brain regions, prompting the massive release of catecholamines from autonomic nerve terminals, thereby damaging cardiomyocytes and inducing arrhythmias or MI [7]. A recent study using stereotactic electrode technology first mapped the human brain topography for cardiac rhythm regulation, expanding the classical scope of the brain–heart interaction. Cardiac pulsation-specific activation occurs not only in the insula, amygdala, and anterior cingulate gyrus but also in the dorsolateral prefrontal cortex, supramarginal gyrus, and superior temporal gyrus [8].

Metabolism plays bidirectional regulatory roles between the brain and the heart and involves the construction of a complex interactive network through energy substrate competition and cross-organ transmission of signal molecules. Brain-derived substances can regulate cardiac function, and changes in cardiac function can also affect brain function through metabolites. Studies have shown that patients with mental illnesses are prone to cardiovascular comorbidities due to metabolic disorders, with rapid elevation of blood lipid levels in the early stage [9]. Besides direct neural regulation, metabolic factors also mediate the intricate crosstalk between the brain and the heart. Plasma catecholamine levels are positively associated with myocardial injury in ischemic stroke patients [10]. Furthermore, elevated interleukin-6 (IL-6) and tumor necrosis factor-alpha (TNF-α) in heart failure (HF) patients with depression and cognitive impairment also indicate that inflammation contributes to brain-heart interactions [11], whereas subarachnoid hemorrhage can induce inflammatory cell infiltration in the heart through excessive activation of the sympathetic nerve, indicating that inflammatory factors may mediate myocardial injury after brain injury [12]. Taken together, brain injury-induced metabolic disorders exacerbate cardiac functions, possibly through endocrine or inflammatory factor pathways.

On the other hand, cardiac dysfunctions also cause brain damage. Studies have shown that cardiac arrest induces abnormal brain glucose metabolism in mice [13] and significantly elevates brain injury markers—the neurofilament light chain and Tau protein—in patients [14,15]. Therefore, cardiac dysfunction might alter brain functions through neural, endocrine, immune, and other mechanisms.

The autonomic nervous system is central to brain–heart interaction. The brain regulates cardiac function through sympathetic and parasympathetic nerves [16], the former increases heart rate and myocardial contractility, while the latter maintains resting heart rate balance. The central autonomic network composed of the anterior insula, anterior cingulate gyrus, and amygdala is crucial for controlling heart rate and myocardial contractility [17]. Conversely, cardiac baroreceptors affect the balance of the central nervous system through feedback signals [18]. Metabolically abnormal neurotransmitters can affect cardiac function, and chronic sleep imbalance can induce autonomic dysfunction and exacerbate hypertension and tachycardia via norepinephrine (NE) release [19].

In conclusion, the brain and heart form a complicated interactive network. Metabolic homeostasis is the foundation for the synergic responses between the brain and the heart, and metabolic disorders constitute the core mechanism underlying brain-heart comorbidities. The mechanisms of metabolic interactions between the brain and heart urgently need to be clarified, and cross-organ protection strategies targeting metabolic nodes are urgently needed to provide innovative therapeutic approaches for brain-heart comorbidities, such as stroke, HF, and diabetes.

## 2. Brain Metabolism Regulates Cardiac Functions

### 2.1. Neurometabolic Regulation

Neurotransmitters in the brain not only mediate signal transmission between neurons, but also directly regulate cardiac function through autonomic neural pathways. This section discusses the roles of four representative metabolites—glutamate (Glu), NE, dopamine (DA), and arachidonoylethanolamide (AEA)—in brain–heart regulation. Although these substances act on distinct brain regions and receptors, they share a common regulatory pattern: they mainly modulate heart rate, myocardial contractility, and cardiac inflammatory status by regulating the sympathetic–vagal balance. The following section will elaborate on the signaling pathways of each metabolite separately and compare their similarities and differences at the end of the subsection.

#### 2.1.1. Glu

As one of the most important excitatory neurotransmitters in the central nervous system, Glu is widely distributed in multiple brain regions, such as the cerebral cortex, hypothalamus, and brainstem. It forms a neural signal transmission network by binding to Glu receptors, thereby regulating cardiac function. However, under stress conditions, the structure and function of glutamatergic neural circuits in the brain are altered, which can lead to disorders in cardiovascular regulatory function and induce cardiac functional abnormalities, such as arrhythmia.

Glu regulates cardiac function through multiple descending pathways originating from cortical and subcortical structures, all converging on brainstem autonomic centers. Three distinct glutamatergic circuits have been identified. First, in the motor cortex–raphe pathway, activating glutamatergic neurons (GluN) in the primary motor cortex (M1) of MI mice increases heart rate and low-frequency to high-frequency power (LF/HF) ratio while decreasing left ventricular ejection fraction; this effect is mediated by the median raphe nucleus (MnR), as muscarinic inhibition of MnR abolishes the cardiac response. Notably, this regulatory function is specific to M1, as activating GluN in secondary motor or sensorimotor cortex produces no cardiac effects [20]. Second, in the cortico-thalamo-hypothalamic pathway, optogenetic stimulation of the anterior cingulate cortex (ACC) activates a descending chain, ACC–ventromedial thalamus–dorsomedial hypothalamus (DMH)–nucleus ambiguous, which mediates operant bradycardia in rats [21]. Third, in the habenula–medulla pathway, a glutamatergic connection from the medial habenula (MHb) to the rostral ventrolateral medulla (RVLM) regulates cardiovascular function in a posttraumatic stress disorder model; MHb stimulation increases RVLM firing and heart rate, effects blocked by RVLM Glu receptor antagonism [22]. Collectively, these pathways demonstrate that cortical and limbic GluN populations can independently influence cardiac function by engaging distinct brainstem autonomic outputs. See the entries related to Glu in Table 1.

#### 2.1.2. NE

NE neurons in two brainstem nuclei—the nucleus tractus solitarius (NTS) and the locus coeruleus (LC)—regulate cardiac function by modulating autonomic balance. In the NTS, A2 NE neurons integrate cardiovascular afferent signals and adjust sympathetic–vagal balance. Destroying these neurons in rats subjected to chronic stress increases the LF/HF ratio, indicating enhanced sympathetic tone. Thus, NTS-derived NE contributes to autonomic adaptation during sustained stress [23].

In the LC, the brain’s primary NE synthesis site [24], activating NE neurons inhibits cardiac vagal neurons (CVNs) via increased inhibitory postsynaptic currents. This disinhibition of sympathetic output raises heart rate and myocardial contractility to meet metabolic demands during stress, but also elevates the risk of arrhythmias [25].

Together, NTS and LC NE neurons form complementary brainstem hubs: the NTS integrates reflex signals, while the LC coordinates global stress responses—both ultimately tuning the sympathetic–vagal balance to influence cardiac function. See the entries related to NE in Table 1.

#### 2.1.3. DA

DA is an important catecholamine among neurotransmitters and plays a significant role in regulating cardiac functions. DA can precisely regulate the heart rate and maintain the frequency of heartbeats. In addition, it can also control the contractile and diastolic abilities of the heart.

Activation of dopaminergic neurons in the ventral tegmental area (VTA)—a core hub of the brain’s reward system—improves cardiac recovery after MI through pleiotropic effects on inflammation, fibrosis, and remodeling. In MI mice, chemogenetic activation of VTA dopaminergic neurons increases left ventricular ejection fraction and stroke volume while reducing end-systolic and end-diastolic volumes. These functional improvements are accompanied by reduced myocardial fibrosis and scar area, increased vascular density in the damaged myocardium, and decreased plasma levels of pro-fibrotic cytokines IL-6 and transforming growth factor-β(TGF-β). Additionally, VTA activation shifts the cardiac immune landscape by increasing CD4^+^ T cells and decreasing CD8^+^ T cells, promoting a reparative milieu. Thus, VTA dopaminergic signaling represents a central reward-circuit mechanism that remotely influences post-MI cardiac remodeling and repair [26].

The lateral habenula (LHb) regulates cardiovascular function through VTA dopaminergic neurons. When the bilateral VTA is inactivated, electrical stimulation of the LHb—which normally induces bradycardia—paradoxically produces periodic tachycardia. This shift indicates that the VTA is a required relay for LHb-evoked autonomic cardiovascular responses. Thus, LHb signals do not directly project to peripheral autonomic centers but instead engage VTA dopaminergic neurons to mediate heart rate regulation [27]. See the entries related to DA in Table 1.

#### 2.1.4. AEA

Endocannabinoid AEA is produced from phospholipids in neuronal cell membranes via phospholipase A2 and arachidonic acid (AA), and can be hydrolyzed and inactivated by fatty acid amide hydrolase (FAAH). The endocannabinoid system comprises synthetic and metabolic enzymes, cannabinoid receptor type (CB)1 receptors distributed in the central nervous system and peripheral nerve endings, and CB2 receptors located in immune cells [28]. The concentrations of AEA and 2-arachidonoylglycerol (2-AG) are regulated by hydrolases; AEA is mainly hydrolyzed by FAAH, while 2-AG is primarily degraded by monoacylglycerol lipase (MAGL). AEA is directly involved in the regulation of cardiac function.

AEA in the dorsal periaqueductal gray (dPAG) regulates heart rate by modulating sympathetic–parasympathetic balance. AEA injection into the dPAG increases LF/HF ratio, sympathetic activity, and baseline heart rate, while decreasing parasympathetic tone. Higher heart rate correlates with increased AEA levels and reduced FAAH/MAGL expression in the dPAG. AEA levels in the dPAG positively regulate baseline heart rate by modulating sympathetic–vagal activity [29].

Furthermore, in hyperalgesia, increased FAAH activity in the dPAG enhances AEA hydrolysis, leading to opposite cardiac effects. The hyperalgesia group shows lower cannabinoid content but higher FAAH activity in the dPAG. FAAH activity positively correlates with hyperalgesia incidence. Enhanced AEA hydrolysis increases parasympathetic tone and reduces heart rate. AEA increases heart rate via sympathetic activation; this effect is reversed by elevated FAAH activity [30].

Taken together, the four neurometabolites share a common feature: whether it is the excitatory transmitter Glu, the catecholamines NE and DA, or the endocannabinoid AEA, their regulation of the heart ultimately converges on the sympathovagal balance of the autonomic nervous system. Glu enhances sympathetic output mainly through the cortex–brainstem pathway; NE, originating from LC and NTS, bidirectionally modulates cardiac autonomic tone; DA exerts cardioprotective effects via the reward system of VTA; and AEA influences heart rate in PAG by modulating sympathovagal balance. Notably, the same metabolite can even exert opposite cardiac effects in different brain regions, suggesting that brain–heart regulation is highly brain region-specific. See the entries related to AEA in Table 1.

### 2.2. Endocrine Metabolic Regulation

Unlike the rapid and direct effects of neurotransmitters, endocrine–metabolic regulation mediates long-distance signal transmission between the brain and the heart via blood-borne hormonal factors secreted by the hypothalamus, constituting another important metabolic regulatory pathway. As the core hub of neuroendocrine regulation, the hypothalamus participates in the regulation of heart rate, myocardial contractility, and myocardial remodeling by secreting various hormonal factors. In addition to the classic HPA axis, non-traditional endocrine factors such as orexin (OX), oxytocin (OXT), and prostaglandin E2 (PGE2) also play key roles in brain–heart regulation.

#### 2.2.1. OX

OX is a class of neuropeptides secreted by the lateral hypothalamic area. It participates in the regulation of cardiac activities and the maintenance of homeostasis through interactions with specific receptors widely distributed in the central and peripheral nervous systems.

After activation, hypothalamic orexinergic neurons in the RVLM and amygdala release OX, which interacts with receptors to participate in the regulation of cardiac function. Studies have shown that after injecting OX into the RVLM and DMH, RVLM neurons depolarize and induce a significant increase in heart rate. However, when the RVLM and DMH are treated with OX receptor antagonists, the increase in heart rate was considerably inhibited, indicating that OX acts on OX receptors in the RVLM and DMH to regulate heart rate [31,32]. Similarly, after injecting OX into the central amygdala and dorsal medullary nucleus, the heart rate of mice also increased [33,34]. In addition, when gamma-aminobutyric acid antagonists are injected into the amygdala and bed nucleus, the increase in heart rate in OX-deficient mice is reversed, indicating that OX in the hypothalamus is involved in amygdala- or bed nucleus-mediated changes in heart rate [35]. See the entries related to OX in Table 1.

#### 2.2.2. OXT

The paraventricular nucleus (PVN) of the hypothalamus is one of the core regions for neuroendocrine regulation. It can integrate signals such as somatic stress to regulate systemic physiological functions by secreting neuropeptides. As a representative neuropeptide secreted by the PVN, OXT is closely associated with the cardiovascular system.

Activation of OXT neurons in PVN improves cardiac systolic and diastolic functions by protecting myocardial mitochondrial function, reducing the levels of inflammatory factors and chemokines, and decreasing collagen III deposition. In a rat model of pressure overload-induced myocardial hypertrophy, selective activation of OXT neurons promotes OXT release, restores cardiac output, ejection fraction, fractional shortening, and stroke volume, while reducing cardiac collagen III and interleukin-1β (IL-1β) levels to alleviate cardiac injury [36]. In MI models, specific activation of OXT neurons in the PVN enhances excitatory postsynaptic currents in the dorsal motor nucleus of the vagus nerve, protects myocardial mitochondrial function, reduces the levels of IL-1β, the chemokines C-C Motif Chemokine Ligand (CCL) 3 and CCL4, and significantly decreases the incidence of arrhythmias and improves the survival rate of model animals [37].

This further confirms that OXT, as a key endocrine factor derived from the hypothalamus, plays an important bridging role in the brain–heart metabolic regulatory network. See the entries related to OXT in Table 1.

#### 2.2.3. PGE2

PGE2, which is generated from AA by cyclooxygenase, is involved in the regulation of cardiac function. PGE2 is widely distributed in the central nervous system and peripheral organs. In the central nervous system, PGE2 regulates pain sensitivity and promotes the development of neuropathic pain. In peripheral organs, such as the kidney, PGE2 is involved in renal fibrosis and water-salt balance [38]. PGE2 produced in the duodenum and stimulated by hydrochloric acid, is involved in functional dyspepsia [39]. In the cardiovascular system, PGE2 can participate in pathological processes such as arrhythmia and hypertension [40]. In addition, PGE2 in the hypothalamus may be involved in the regulation of the cardiovascular system.

Hypothalamic astrocytes contain PGE2 synthases and receptors. Upon stimulation, they rapidly synthesize PGE2, which acts on adjacent neurons through autocrine/paracrine pathways, activates the HPA axis, enhances myocardial contractility, and accelerates heart rate to meet metabolic demands under stress. However, after ischemia/reperfusion or stress-induced brain injury, excessive production of PGE2 by astrocytes leads to cardiac dysfunction, whereas inhibiting its synthesis or related enzyme activities alleviates cardiac injury. Exogenous stimuli such as nesfatin-1 and galanin-like peptide can induce cyclooxygenase-2 expression in the hypothalamus, promote PGE2 release, and cause tachycardia [41,42]. Studies on key regulatory brain regions have shown that injection of PGE2 into the hypothalamic preoptic area induces tachycardia, and this effect can be reversed by administration of cyclooxygenase inhibitors into the dorsomedial hypothalamic nucleus [43], suggesting that the dorsomedial hypothalamic nucleus serves as an important hub for PGE2-mediated cardiac regulation. Furthermore, the cardiac effects of PGE2 may be mediated by the HPA axis, as it elevates plasma adrenocortical hormone levels and simultaneously increases heart rate and mean arterial pressure [44,45].

In summary, as a key endocrine and metabolic factor derived from hypothalamic glial cells, PGE2 assists the heart in adapting to stress under physiological conditions. However, its excessive release after brain injury mediates cardiac dysfunction through the HPA axis and specific hypothalamic nuclei, making it an important hub in the brain–heart endocrine and metabolic regulatory network. See the entries related to PGE2 in Table 1.

### 2.3. Immunometabolic Regulation

TNF-α is a central inflammatory factor that has a direct regulatory effect on the autonomic nervous system. TNF-α in the PVN of the hypothalamus links brain inflammation and cardiac injury. Intracerebroventricular injection of TNF-α can transiently increase the heart rate and arterial pressure of rats [46]. After bilateral injection of scrmRNA into the PVN of rats with MI, TNF-α levels in the PVN obviously increased, and the left ventricular ejection fraction of the rats decreased. In addition, the left ventricular end-diastolic volume and left ventricular end-systolic volume increased, resulting in the deterioration of cardiac function. However, after bilateral injection of tumor necrosis factor-α converting enzyme simRNA, TNF-α levels in the PVN significantly decreased, and cardiac function injury was alleviated [47].

**Figure 1:**
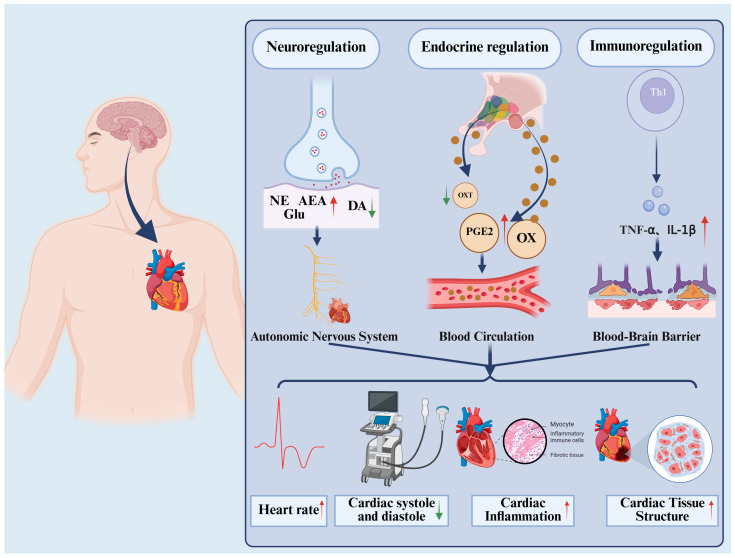
Metabolites in the brain can affect cardiac structure and function through neural, endocrine, and immune pathways. In terms of neural regulation, brain metabolites include DA, NE, Glu, and AEA; in terms of endocrine regulation, brain metabolites include OX, OXT, and PGE2; in terms of immune regulation, brain metabolites include TNF-α. The metabolites involved in the three aforementioned aspects affect heart rate, cardiac contractile function, cardiac inflammation, and changes in cardiac tissue structure through the autonomic nervous system, blood circulation, and disruption of the blood–brain barrier, respectively. DA, dopamine; Glu, Glutamic acid; NE, noradrenergic; AEA, arachidonylethanolamine; OX, orexin; OXT, oxytocin; PGE2, prostaglandin E2; TNF-α, tumor necrosis factor-alpha; IL-1β, interleukin-1β. Created in BioRender. Lzm, L. (2026) https://BioRender.com/mn1yu39, accessed on 20 March 2026.

Current studies have demonstrated the important role of metabolism in brain-heart regulation, explaining the regulation of cardiac function by the aforementioned brain regions from three aspects: neural, endocrine, and immune (Figure 1). On the basis of a literature review, the main brain regions involved in brain-heart regulation and the cardiac functions they affect are shown in Table 1.

**Table 1:** Main brain regions involved in brain-heart regulation and the cardiac functions they affect.

Species	Metabolite	Brain Regions Regulating Heart	Affect Cardiac Function	References
Mice	Glu	M1	Heart rate ⬆ Autonomic nerve balance ⬆	Bo, W., et al., 2024 [20]
		MnR		
Rat		ACC	Heart rate ⬆	Yoshimoto, A., et al., 2024 [21]
		VMT		
		DMH		
		Amb		
Rat		RVLM		
		MHb		
Rat	NE	NTS	Autonomic nerve balance ⬆	Bundzikova-Osacka, J., et al., 2015 [23]
Mice		LC	Heart rate ⬆ Cardiac contractility ⬇	Wang, X., et al., 2014 [25]
Mice	DA	VTA	Cardiac inflammation ⬆ Contractility ⬇ Heart rate ⬆	Haykin, H., et al., 2024 [26]; Sato, Y., et al., 2024 [27]
Rat	AEA	PAG	Heart rate ⬆ Autonomic nerve balance ⬆	Dean, C., et al., 2016 [29]; Dean, C., et al., 2017 [30]
Mice/Rat	OX	Hypothalamus	Heart rate ⬆	Zhang, W., et al., 2009 [35]
Rat	PGE2			Kageyama, H., et al., 2013 [42]
	OXT	PVN	Cardiac inflammation ⬆ Contractility ⬇	Schunke, K.J., et al., 2023 [37]

## 3. Cardiac Function Regulates Brain Metabolism

Cardiac dysfunction not only impacts peripheral hemodynamics but also induces profound metabolic alterations in the brain. Clinical and experimental evidence indicates that HF, MI, and cardiac arrest can disrupt the blood–brain barrier and reprogram cerebral metabolic profiles [48]. These changes span multiple biochemical categories, including carbohydrates, lipids, energy-related metabolites, amino acids, and cytokines, ultimately contributing to cognitive decline, neuronal damage, and neuroinflammation. This section systematically reviews how cardiac injury influences brain metabolism across these five metabolite classes.

### 3.1. Carbohydrate Metabolites

Both clinical and animal studies have shown that HF can cause abnormal glucose metabolism in the brain and changes in neuronal function, which subsequently lead to cognitive and behavioral deficits, with varying responses across different brain regions.

Clinical evidence indicates that HF and myocardial ischemia reduce cerebral glucose metabolism in a region-selective manner, predominantly affecting areas involved in autonomic regulation and memory. In male patients with myocardial ischemia, reduced left ventricular ejection fraction correlates positively with decreased fluorine-18-fluorodeoxyglucose (^18^F-FDG) uptake in the corpus callosum, caudate nucleus, and brainstem [49]. Similarly, HF patients show ^18^F-FDG hypometabolism in the bilateral occipital cortex, caudate nucleus, thalamus, hippocampus, frontal lobe, and posterior cingulate cortex, accompanied by volume loss in the thalamus and hippocampus [50]. Importantly, the extent of hippocampal and thalamic atrophy correlates negatively with plasma N-terminal pro-brain natriuretic peptide levels, directly linking cardiac dysfunction severity to cerebral metabolic decline. Together, these clinical findings suggest that the hippocampus and thalamus—hubs for memory and autonomic integration—are particularly vulnerable to cardiac injury-induced hypometabolism.

Animal studies complement clinical observations by revealing temporal dynamics and direct cognitive consequences of HF-induced glucose dysmetabolism. HF mice exhibit behavioral deficits—including impaired exploratory behavior, short-term memory, and spatial learning—along with hippocampal endoplasmic reticulum Ca^2+^ leakage and impaired long-term potentiation [51]. The direction of metabolic change depends on disease phase: acute HF rats show increased glucose metabolism in the prefrontal and cingulate cortices, whereas chronic HF rats display reduced metabolism in these same regions, accompanied by hippocampal Cornu Ammonis 1 (CA1) neuronal degeneration and cognitive impairment [52]. Furthermore, regional sensitivity to cardiac arrest-induced ischemia varies considerably: glucose levels drop sharply in frontal, parietal, occipital, and temporal cortices within 2 h, but remain unchanged in striatum, hippocampus, thalamus, brainstem, and cerebellum [53]. This regional and temporal heterogeneity suggests that compensatory metabolic activation in acute stages may eventually fail, leading to chronic hypometabolism and cognitive decline.

### 3.2. Lipid Metabolites

Cardiac injury can significantly affect lipid metabolism in the brain. Studies have shown that linoleic acid levels in the brain are markedly decreased as early as 20 min after cardiac arrest, and both linoleic acid and linolenic acid are further reduced 30 min after resuscitation, suggesting that cardiac dysfunction can rapidly disrupt the homeostatic balance of cerebral lipid metabolism [54].

As a unique phospholipid in brain mitochondria, cardiolipin plays an important role in cerebral ischemia–reperfusion (IR) injury mediated by cardiac arrest. Cardiopulmonary resuscitation after cardiac arrest can increase cardiolipin levels in the rat heart and exacerbate cerebral ischemic injury. Notably, cardiolipin is decreased in the hippocampus but increased in plasma, and its changes are positively correlated with the duration of cardiac arrest, indicating that hippocampal cardiolipin can be released into the blood during cardiac arrest and participate in brain injury [55]. Further studies have demonstrated that cognitive impairment following cardiopulmonary resuscitation is closely associated with lipid metabolism disorders, characterized by significant accumulation of phosphatidylcholine and phosphatidylethanolamine in the hippocampal CA1 region, accompanied by reduced calcium-independent phospholipase A2 activity. Exogenous supplementation with docosahexaenoic acid can improve postoperative cognitive function, enhance phospholipase A2 activity, and alleviate lipid accumulation in the hippocampus [56]. Collectively, these results indicate that cardiac injury can induce abnormal lipid deposition in the hippocampus, thereby leading to brain damage.

### 3.3. Energy Metabolism-Related Substances

Cardiac injury can induce cerebral energy metabolic disturbances and mitochondrial dysfunction. Myocardial IR injury reduces the expression of mitochondrial fusion proteins in the whole brain, leading to imbalanced mitochondrial dynamics and dysfunction; pretreatment with the mitochondrial fusion promoter can alleviate these injuries and reduce brain damage [57]. MI mediates abnormal mitochondrial crosstalk between the heart and brain through inflammatory signals: mitochondrial transporters are increased in the infarcted area and colocalized with CD48 inflammatory cells, accompanied by elevated levels of the same transporters in microglia of the cerebral cortex. Enalapril treatment can reverse these changes, suggesting that mitochondrial transport is involved in the remote activation of microglia in the brain by inflammation [58]. Furthermore, cardiopulmonary resuscitation after cardiac arrest significantly reduces cerebral adenosine triphosphate (ATP) and acetyl-CoA levels, resulting in energy metabolism disorders. Overexpression of acetyl-CoA synthetase 2 in brain microvascular endothelial cells can improve endothelial dysfunction and protect blood vessels by regulating transcription factor EB and the AMPKα pathway, thereby alleviating brain injury [59]. These findings reveal the key mechanisms by which cardiac injury causes brain damage through affecting cerebral energy metabolism, and provide potential targets for the development of related therapeutic strategies.

### 3.4. Amino Acid Metabolites

Cardiac injury not only affects carbohydrate and lipid metabolism but also induces brain damage through amino acid metabolic pathways. As they are neurotransmitter precursors and substrates for energy metabolism, the imbalance of amino acids in the brain is one of the important mechanisms underlying cardiogenic brain injury.

Studies have shown that cardiac surgery can cause mild damage to rat hippocampal neurons, while chronic unpredictable stress significantly exacerbates hippocampal neuronal degeneration. Metabolomic analysis suggests that amino acid metabolic disturbance is the main cause, in which abnormal lysine degradation and β-alanine metabolism may play key roles [60]. In addition, selective overexpression of adenylate cyclase in the heart can induce tachycardia, upregulate the expression of DA receptor 5, GABA-A receptor, and metabotropic Glu receptor 1/5 in the hippocampus, and enhance locomotor activity in mice [61].

To clarify the dynamic changes in cerebral amino acid metabolites after cardiac arrest, researchers subjected rats to 30 min of cardiopulmonary bypass resuscitation following 20 min of cardiac arrest. The results showed that most amino acids were increased after resuscitation, whereas aspartic acid and glutamic acid were decreased, indicating that cardiac arrest can significantly alter the cerebral amino acid metabolic profile [54]. Further analysis of different brain regions revealed that taurine levels were elevated in the parietal cortex, temporal cortex, striatum, hippocampus, and thalamus after cardiac arrest; choline levels were decreased in the midbrain and medulla-pons, but increased in the thalamus. These findings demonstrate that cardiac pathological conditions can differentially regulate amino acid metabolism in distinct brain regions [62].

In summary, cardiac injury can induce disturbances in cerebral amino acid metabolism, manifested as region-specific changes in the levels of various amino acids. Imbalanced amino acid metabolism is not only an important feature of cardiogenic brain injury but may also be involved in the development of cognitive dysfunction by affecting neurotransmitter synthesis and energy metabolism. Targeting amino acid metabolic pathways is expected to become a potential interventional strategy for alleviating brain damage after cardiac surgery.

### 3.5. Cytokines

Cardiac injury can induce cytokine-mediated neural damage by activating inflammatory responses in the brain. Cardiac events such as MI and cardiac arrest can trigger the activation of glial cells and the release of related inflammatory factors in the brain, participating in the pathological process of cardiogenic brain injury.

Studies have shown that following MI and IR injury, left ventricular ejection fraction is reduced, while microglia and astrocytes in the hippocampal CA1 region are activated, and IL-1β levels are elevated. Administration of the apoptosis inhibitor Z-VAD can improve cardiac dysfunction, suppress excessive glial activation, and alleviate hippocampal inflammation by reducing IL-1β levels, suggesting that cardiac injury can induce inflammatory responses in the brain [63].

In addition, upon restoration of spontaneous circulation after cardiac arrest, rat survival rate is decreased, apoptotic cells in the hippocampal CA1 region are significantly increased, and mRNA expression of TNF-α and IL-1β is upregulated in both the hippocampus and cortex. Intraperitoneal injection of dichloroacetate, a pyruvate dehydrogenase kinase inhibitor, improves survival, reduces cellular apoptosis, and lowers the levels of the aforementioned inflammatory factors, indicating that activation of apoptotic pathways and release of inflammatory factors jointly contribute to brain injury after cardiac arrest [64]. Thus, cardiac injury events such as MI and cardiac arrest can induce inflammatory responses in the brain, characterized by the activation of glial cells and elevated levels of inflammatory factors, including IL-1β and TNF-α. These changes are closely associated with neuronal apoptosis in the hippocampal region and cognitive dysfunction.

**Figure 2:**
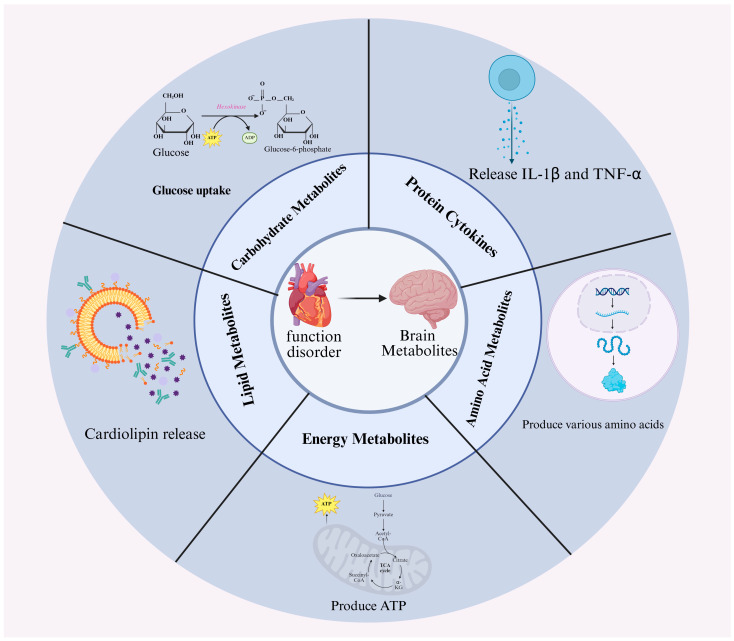
Under conditions of impaired cardiac function, metabolic disorders occur in brain metabolites. Regarding carbohydrate metabolites, the glucose uptake in the brain decreases; regarding lipid metabolites, the release of cardiolipin in the brain increases, accompanied by abnormal lipid accumulation; regarding energy metabolism, the release of ATP and acetyl-CoA in the brain decreases, with abnormal mitochondrial function; regarding amino acid metabolism, fluctuations are observed in amino acid metabolites throughout the brain; regarding cytokines, the release of TNF-α and IL-1β in the brain increases. Created in BioRender. Lzm, L. (2026) https://BioRender.com/g5tcdrz, accessed on 20 March 2026.

In summary, cardiac dysfunction triggers widespread metabolic disturbances in the brain, affecting glucose utilization, lipid homeostasis, energy production, amino acid balance, and inflammatory cytokine expression (As shown in Figure 2, the effects of cardiac dysfunction on brain metabolism include five categories of substances). These metabolic alterations are region-specific and closely associated with neuronal injury and cognitive impairment. Understanding these cardiac-driven cerebral metabolic changes provides a crucial foundation for uncovering the mechanisms of brain–heart comorbidity and identifying potential metabolic targets for neuroprotection in patients with heart disease.

## 4. Bidirectional Regulation of Brain–Heart Metabolism

The interaction between the heart and brain is not unidirectional; instead, they form a complex bidirectional regulatory network through metabolic, immune, and neural pathways. Cardiac injury can induce inflammation and functional alterations in the brain, whereas interventions targeting the brain can, in turn, affect cardiac prognosis. This bidirectional regulatory mechanism plays a critical role in maintaining the homeostasis of bodily functions.

Currently, bidirectional regulation between the heart and brain has attracted increasing attention. For example, brain injury is induced in MI models; intervention in brain injury can also regulate cardiac injury, and metabolic regulation is an important mechanism. It has also been reported that MI induces brain inflammation, and subsequent brain intervention can affect cardiac prognosis. Therefore, the bidirectional regulation of brain–heart metabolism is an important mechanism for maintaining the homeostasis of body functions.

Studies have shown that microglia are activated in the brains of MI model mice and that monocytes from the heart are recruited to the lateral posterior nucleus (LPN) of the thalamus through the choroid plexus, increasing the pressure and abundance of rhythmic slow-wave sleep. Moreover, knockdown of the TNF receptor TNFR1 on GluN in the thalamic LPN weakens slow-wave sleep pressure. Taken together, these findings suggest that monocytes are recruited to the thalamic LPN and produce TNF, enabling GluN expressing Tnfrsf1a to participate in the sleep process after myocardial injury. On the other hand, sleep fragmentation increases the number of macrophages and chemokines CCL3 and CCL4 in the heart, reduces heart rate variability, increases cardiac autonomic sympathetic input, and finally leads to cardiac inflammation and spontaneous arrhythmias. Moreover, Tnfrsf1a knockdown in GluN of the thalamic LPN results in increased DA β-hydroxylase and NE in the heart, upregulates β-adrenergic receptors in macrophages, and promotes the accumulation of monocytes and macrophages, which exacerbates cardiac injury. In contrast, TNF stimulation in the thalamic LPN increases sleep and ameliorates cardiac activity. In conclusion, uninterrupted sleep after MI can reduce sympathetic input to the infarcted heart, thereby reducing monocyte recruitment and reducing cardiac inflammation, suggesting that regular sleep may improve cardiac prognosis through sympathetic–immune interactions [65].

In MI model rats, the left ventricular ejection fraction decreases, the left ventricular end-diastolic volume increases, and cardiac function is impaired, which significantly increases the levels of interleukin-17A (IL-17A) in cerebrospinal fluid and serum and significantly increases the levels of IL-17A and its receptor IL-17RA in the PVN. In the PVN of MI model rats, IL-17RA knockdown notably reduced the levels of TNF-α, IL-1β, IL-6, monocyte chemoattractant protein-1, and macrophage inflammatory protein-1α; decreased the number of c-Fos-positive cells in the PVN; and reduced the NE concentration in the serum. These findings indicate that IL-17RA knockdown can reduce the levels of inflammatory mediators and inhibit sympathetic nerve activity in the PVN. In addition, studies have shown that IL-17RA knockout in the PVN inhibits the decrease in the left ventricular ejection fraction and the increase in the left ventricular end-diastolic volume, thereby improving cardiac insufficiency. In conclusion, impaired cardiac function increases IL-17A levels in the PVN and activates sympathetic nerves, which results in persistent cardiac damage [66].

In conclusion, the bidirectional regulation of heart–brain metabolism involves multiple processes, including immune cell recruitment, sleep modulation, inflammatory factor release, and sympathetic nerve activation. Cardiac injury can act back on the heart itself through intracerebral inflammation and neural pathways, forming positive or negative feedback regulatory loops. A thorough understanding of the heart–brain bidirectional regulatory mechanisms, particularly the interactions among sleep, inflammation, and sympathetic activity, will provide novel intervention targets and strategies for the prevention and treatment of cardio–cerebrovascular comorbidities.

## 5. Summary and Prospects

With the rapid development of systemic biology, research on brain–heart interaction has attracted increasing attention. Most studies focus on the unidirectional regulation of the heart by the brain via neural, endocrine, and immune pathways under stress. However, the reverse effects of the heart on brain function, especially metabolite fluctuations caused by cardiac injury, remain less investigated. Studies have shown that cardiac injury disturbs brain metabolism, including increased glucose uptake, lipid accumulation, reduced ATP levels, abnormal amino acid metabolism, and inflammatory factor release. Metabolic disorders are common in brain–heart comorbidities: brain injury increases cardiovascular risk, while heart disease impairs brain function. Brain-heart metabolic interactions are bidirectionally regulated, including heart-brain-heart feedback and negative feedback mechanisms.

Besides the direct bidirectional metabolic regulation mentioned above, the heart and brain can also interact indirectly through other organs. Activation of dopaminergic neurons in the VTA of the brain upregulates hepatic complement 3 expression, thereby improving cardiac function, reducing myocardial fibrosis and scar formation, and promoting cardiac repair after MI [26]. Another study has proposed the heart–brain–spleen axis: under hypertensive stress, the heart activates relevant nuclei in the brain, which stimulates the spleen to release placental growth factor via the sympathetic nerve, thereby promoting the proliferation of cardiac macrophages and helping the heart adapt to pressure overload [67].

Advances in brain science have enabled the precise localization of the regulation of the heart by brain regions, but traditional methods for heart rate control are invasive. The newly developed noninvasive optical pacemaker can be used to study the function of relevant brain regions and provide technical support for exploring the effects of metabolites in brain areas on cardiac function [68].

The brain regulates cardiac activities through neural and humoral pathways, while the heart, in turn, affects brain function via multiple mechanisms. Studies on brain–heart interactions have theoretically integrated these two organs into a coordinated dynamic system, advancing life sciences. Clinically, monitoring brain–heart interactions enable early diagnosis and personalized therapy for cardio-cerebral diseases, improving prognosis. Thus, brain–heart interaction research is of great theoretical and clinical significance and holds transformative potential.

As an interdisciplinary frontier between neuroscience and cardiovascular medicine, brain–heart interaction has achieved important progress. However, several key issues remain: the causal relationship or common pathological basis of brain and heart diseases is unclear; unified diagnostic criteria and large-scale clinical trials are lacking; and the underlying signaling molecules and pathways remain poorly understood. In the future, combining artificial intelligence and multi-omics to construct a brain–heart metabolic network model is expected to predict therapeutic targets and achieve simultaneous treatment of brain and heart with a single drug.

## Data Availability

No new data were created or analyzed in this study. Data sharing is not applicable to this article.

## Abbreviations

MI, myocardial infarction; HPA, hypothalamic-pituitary-adrenal; IL-6, interleukin-6; TNF-α, tumor necrosis factor-alpha; HF, heart failure; AA, arachidonic acid; AEA, arachidonoylethanolamide; 2-AG, 2-arachidonoylglycerol; FAAH, fatty acid amide hydrolase; CB, cannabinoid receptor type; MAGL, monoacylglycerol lipase; DA, dopamine; Glu, glutamate; GluN, Glutamatergic neurons; LF/HF, low-frequency to high-frequency power; M1, primary motor cortex; MnR, median raphe nucleus; ACC, anterior cingulate cortex; VMT, ventromedial thalamic nucleus; DMH, dorsomedial hypothalamus; Amb, ambiguous; RVLM, rostral ventrolateral medulla; MHb, medial habenula; NE, norepinephrine; NTS, nucleus tractus solitarius; LC, locus coeruleus; CVNs, cardiac vagal neurons; VTA, ventral tegmental area; TGF-β, transforming growth factor-β; LHb, lateral habenula; dPAG, dorsal periaqueductal gray; OX, orexin; OXT, oxytocin; PGE2, prostaglandin E2; PVN, paraventricular nucleus; IL-1β, interleukin-1β; CCL, C-C Motif Chemokine Ligand; BBB, blood–brain barrier; ^18^F-FDG, fluorine-18-fluorodeoxyglucose; IR, ischemia–reperfusion; CA1, cornu Ammonis 1; ATP, adenosine triphosphate; LPN, lateral posterior nucleus; IL-17A, interleukin-17A.
